# Performance of Methyl-5-Benzoyl-2-Benzimidazole Carbamate (Mebendazole) as Corrosion Inhibitor for Mild Steel in Dilute Sulphuric Acid

**DOI:** 10.1155/2020/2756734

**Published:** 2020-06-24

**Authors:** F. O. Edoziuno, A. A. Adediran, B. U. Odoni, M. Oki, P. P. Ikubanni, O. Omodara

**Affiliations:** ^1^Department of Metallurgical Engineering, Delta State Polytechnic, Ogwashi-Uku, Ozoro, Nigeria; ^2^Department of Mechanical Engineering, Landmark University, PMB 1001, Omu-Aran, Kwara, Nigeria; ^3^Department of Metallurgical and Materials Engineering, Federal University of Technology Akure, PMB 704, Akure, Ondo, Nigeria

## Abstract

The inhibitive effect of mebendazole (MBZ) on the corrosion of low-carbon steel in H_2_SO_4_ was investigated by gravimetric and electrochemical techniques as well as examination of specimens in the scanning electron microscope with attached energy dispersive X-ray spectrometer (EDS). From gravimetric analysis, the highest inhibition efficiency of about 96.6% was obtained for 1.0 g of inhibitor in H_2_SO_4_ solution at 24 h, while with longer exposure times of between 72 to 120 h, the efficiencies averaged between 92 and 95%. Tafel extrapolations from the polarization curves showed that 1.0 g MBZ gave a maximum inhibition efficiency of approximately 99% for the investigation conducted at 30°C, whereas 1.5 g of MBZ gave a maximum inhibition efficiency of about 85% at 60°C. Inhibition efficiency increased with increasing concentrations of MBZ and decreased at elevated temperatures. The inhibitive action was attributed to physical adsorption of MBZ species on the mild steel surface which followed the Langmuir adsorption isotherm. MBZ performed as a mixed-type inhibitor on mild steel in dilute H_2_SO_4_.

## 1. Introduction

In order to mitigate corrosion, an effective and flexible means is through the use of corrosion inhibitors [1, 2]. Any chemical or combination thereof that reduces corrosion rates without significant reactions with the components of the environment is an inhibitor [3–5]. Usually all major industries involved in processing of chemicals normally consider the use of inhibitors as first line of defence against aggressive attack on plant equipment. Mild steel finds major application in a wide range of industries because of its excellent physical and mechanical properties, low cost, and availability, but it has very low corrosion resistance in aggressive service environments [6, 7]. A large number of researches have focussed on various organic/inorganic corrosion inhibitors for mild steel in acidic environments with varying inhibition efficiencies [8–15]. Corrosion inhibitors have found major use in closed systems with good circulation and controlled inhibitor concentrations, which may include cooling water recirculation systems, oil production and refining, industrial acid cleaning, and acid pickling of steel components. However, inorganic inhibitors such as chromates and zinc salts have suffered major setbacks in their usage as a result of toxicity and have largely been replaced with organic inhibitors [16, 17]. Therefore, practical selection criteria of corrosion inhibitors from huge varieties of inorganic and organic chemicals which have inhibiting properties do not depend solely on their inhibition efficiencies but also on the safety of use, economic constraints, and compatibility with other chemicals in the system and environmental concerns [18–20]. Some of the most effective organic inhibitors include amines, sulphur compounds such as thioethers, thiourea, thioalcohols, hydrazine and thioamides, and heterocyclic nitrogen compounds [21]. Organic compounds are good inhibitors especially when they have functional electron-rich centres such as pi-electron in triple or conjugated double bonds and the presence of aromatic rings in their structures which serve as major adsorption centres [21–23].

A review of the literature revealed the use of benzimidazole derivatives as promising corrosion inhibitors for steel in various environments [7, 13, 24–31]. Benzimidazole and its derivatives have excellent pharmacological properties such as anti-bacterial, anti-fungal, anti-cancer, anthelminthic, etc. In addition, they display planar structures with anchoring sites at sp^2^ hybridized nitrogen carrying lone pairs of electrons coupled with aromatic rings. These and other conjugating factors make them relatively more suitable for use as environment friendly and biodegradable corrosion inhibitors [26]. The present work investigates the performance of Methyl-5-benzoyl-2-benzimidazole Carbamate (mebendazole) as corrosion inhibitor for mild steel in H_2_SO_4_ using gravimetric analyses and electrochemical polarization techniques in addition to scanning electron microscopy examinations. Mebendazole is a synthetic benzimidazole derivative which may be nontoxic and biodegradable and has been employed in therapeutic treatments as anthelmintic drug that has the molecular formula of C_16_H_13_N_3_O_3_ with a molecular weight of 295.3 g/mol.

## 2. Experimental Techniques

### 2.1. Materials

#### 2.1.1. Test Media

The gravimetric analyses were carried out at ambient temperatures of about 30°C in 1.5 M H_2_SO_4_, prepared with double distilled deionized water. Wormin® Mebendazole tablets, manufactured by CADILA pharmaceutical limited, India, were purchased from NBC pharmaceutical coy at Aladja Warri, Nigeria. The tablets were ground to fine powder to increase the surface area and enhance the rate of corrosion inhibition. 100 ml of the dilute H_2_SO_4_ with or without the addition of specified concentrations of mebendazole (MBZ) inhibitor which ranged from 0 as control through 0.5 to 1.0, 1.5 were utilized.

#### 2.1.2. Test Specimens

Commercially available 16 mm diameter mild steel (rod with composition Fe = 98.224%, C = 0.288%, Mn = 0.624, Cu = 0.268, Cr = 0.182, S = 0.052, P = 0.051, Ni = 0.090, Al = 0.021) was machined to obtain specimens with dimensions 15 mm in thickness and 10 mm length. A 5 mm hole was made at the centre of the electrodes. The samples were mechanically ground with series of emery papers with grades from 800 to 1200. The specimens were rinsed with distilled water, degreased with acetone, and allowed to dry before each corrosion test.

### 2.2. Methods

#### 2.2.1. Gravimetric Measurements

Weighed specimens were totally immersed by suspension in each of the various test media in a 200 ml closed beaker for different contact times of 24, 48, 72, 96, and 120 h in the presence and absence of MBZ inhibitor. After the various immersion periods, the test specimens were retrieved from the corroding systems and subsequently washed with distilled water, rinsed with acetone, air-dried, and re-weighed. Equation (1) [7, 14] describes the relationship among the corrosion rate (CR) in millimetres per year (mm/yr) and other parameters employed in this study:

(1) $$\begin{array}{c} \text{CR}=\frac{87.6\times \text{WL}\times 1000\text{mg}}{D\times A\times T}, \end{array}$$

where WL is the weight loss in grams, *D* is the density in g/cm^3^, *A* is the area in cm^2^, and *T* is the time of exposure in hours. The % inhibitor efficiency, *η*, was calculated using equation (2):

(2) $$\begin{array}{c} \eta =\frac{{\text{CR}}_{c}-{\text{CR}}_{i}}{{\text{CR}}_{c}}\times 100, \end{array}$$

where CR_*c*_ and CR_*i*_ are the corrosion rates in the absence and the presence of inhibitor, respectively.

### 2.3. Electrochemical Measurements

The polarization experiments were carried out using an Autolab potentiostat/galvanostat VersaSTAT 4 Electrochemical System controlled from a computer by a Universal Serial Bus (USB) interface using the Versa Studio electrochemistry software package. A conventional three-electrode Pyrex glass cell was employed for the polarization measurements at 30°C and 60°C, respectively. For each measurement, test specimens, with 1 cm^2^ of exposed areas, were employed as working electrodes with a platinum rod as counter electrode. Ag/AgCl was used as the reference electrode with a luggin probe placed close to the working electrode. All experiments were undertaken in stagnant aerated solutions. The working electrode was immersed in the test solution for one hour until a stable open circuit potential was attained. The potentiodynamic polarization study was conducted at a potential range of −250 mV to +250 mV with respect to the corrosion potential using linear sweep technique at a scan rate of 1 mV/s. The linear Tafel segments of the anodic and cathodic curves were extrapolated to equilibrium potential in order to obtain the corrosion current densities (*i*_corr_). Each experiment was carried out without MBZ inhibitor and with various concentrations of the inhibitor. The inhibition efficiency *η* was calculated using equation (3):

(3) $$\begin{array}{c} \eta =\frac{{\text{icorr}}_{C}-{\text{icorr}}_{i}}{{\text{icorr}}_{C}}\times 100, \end{array}$$

where icorr_*i*_ and icorr_*C*_ are the corrosion current densities of mild steel coupon in the presence and absence of inhibitor, respectively.

### 2.4. Scanning Electron Microscopy/EDS Examination

Phenom Pro *X* Model SEM (Phenom world, Eindhoven, Netherlands) equipped with Energy Dispersive X-ray spectroscopy (EDS) was employed to examine the surface morphology of the mild steel specimens after gravimetric measurements.

## 3. Results and Discussions

### 3.1. Gravimetric Measurements

**Figure 1:**
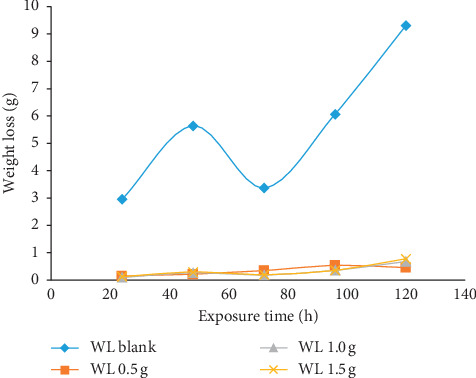
Weight loss variation of mild steel in dilute H_2_SO_4_ with immersion time in the presence of various concentrations of MBZ.

**Figure 2:**
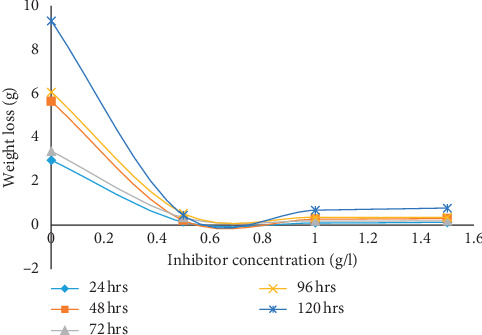
Weight loss (g) variation of mild steel in dilute H_2_SO_4_ with inhibitor concentration (g/l).

**Figure 3:**
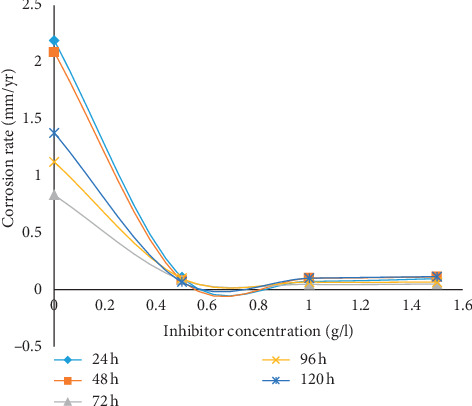
Corrosion rate (mm/yr) of mild steel in dilute H_2_SO_4_ with variation in inhibitor concentration (g/l) at various immersion times.

**Figure 4:**
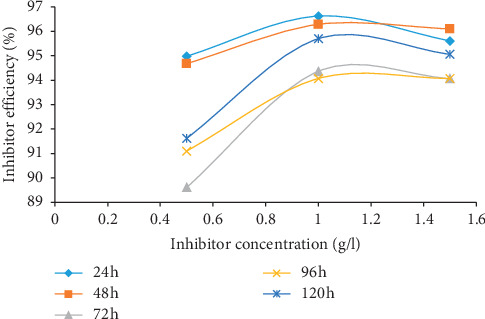
Inhibitor efficiency (%) versus concentration of MBZ for mild steel in 1.5 M H_2_SO_4_.

The results of gravimetric measurement in terms of weight loss (WL), corrosion rate (CR), percentage inhibition efficiency (*η*), and surface coverage (Φ) are displayed in Table 1 while the graphical representations for the various parameters are presented in Figures 1–4. The plot of weight loss against exposure time for mild steel with and without varying concentrations of MBZ (Figure 1) showed significant decrease in weight loss upon the introduction of MBZ into the corrosive media. The rate at which weight loss occurred was reduced with increases in the concentration of the inhibitor (Figure 2). Similar trend was observed in the corrosion rates versus concentration of the inhibitor as displayed in Figure 3. While inhibition efficiency increased with increase in inhibitor concentrations as portrayed in Figure 4.

The range of weight loss (0.10–0.78 g), corrosion rate (0.01–0.12 mm/yr), inhibition efficiency (89.61–96.62%), and surface coverage (0.90–0.97) indicated that MBZ performed creditably well as an inhibitor of acid corrosion of mild steel. This may be attributed to effective and increased adsorption, albeit with increase in time and concentration of inhibitor molecules on the surface of the mild steel test coupons. Thus, the relatively high values of inhibition efficiency and surface coverage and lower values of corrosion rates and weight losses may be a result of the presence of several heteroatoms, such as nitrogen and oxygen atoms as well as the presence of nonbonding *π*-electrons in the ringed structure of MBZ [32]. These heteroatoms and free *π*-electrons provided the electron-rich sites through which MBZ adsorbed severally on the mild steel surface and thereby interfered positively with the anodic and/or cathodic reactions to reduce the corrosion rates of the mild steel.

It can be observed from Table 1 and Figures 1–4 that corrosion rate and weight loss values were reduced to a minimum with increase in concentration of inhibitor and exposure time. The lowest corrosion rate value of 0.01 mm/yr corresponding to a weight loss of 0.13 g was achieved at 1.5 g/l of MBZ over an immersion period of 24 h with a corresponding inhibition efficiency of about 96%.

**Figure 5:**
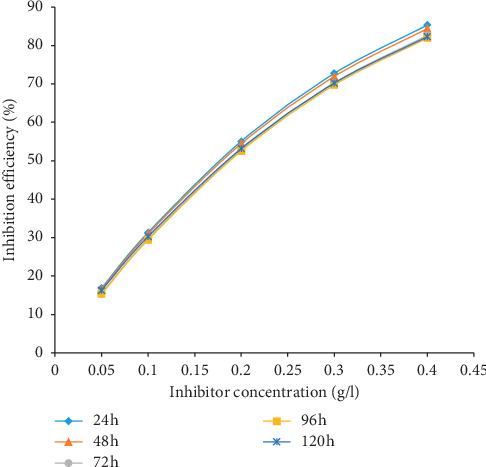
Plot of modelled inhibition efficiency (%) versus concentration of MBZ for mild steel in dilute H_2_SO_4_.

Because of the small differences between the inhibition efficiencies of the highest and lowest inhibitor concentrations, the experimental weight loss results were used to model and predict the performance at lower concentrations of the inhibitor (0.05, 0.1, 0.2, 0.3, & 0.4 g/l). Using Response Surface Methodology (RSM), the inhibition efficiency and other corrosion properties were optimized by central composite design (CCD) tool of Design Expert software version 11.0. A quadratic design model was applied during the analysis. Inhibitor concentration and immersion time were set as the independent variables (Factors 1 & 2), while weight loss (WL), corrosion rate (CR), inhibitor efficiency (IE), and degree of surface coverage (SC) were the response variables (Responses 1 to 4). Thirty runs of experiments were carried out to obtain the responses of the dependent variables/corrosion properties. Predictive model equations for inhibition efficiency (IE) of MBZ, corrosion rate (CR), and weight Loss (WL) as a function of the considered factors in terms of coded factors is given by equations (4a)–(4c)). Nonlinear, Quartic model equations sufficiently described the relationship between inhibition efficiency, corrosion rate, and the independent factors (inhibitor concentration (*B*) and immersion time (*A*), respectively (equations (4a) and (4b)). Conversely, a quadratic model described the relationship between weight loss (WL) and the independent factors (equation (4c)). The detailed experimental design with the graphical plots, mathematical modelling, optimization, analysis of variance (ANOVA), other statistical evaluations, etc. are reported in [33, 34]. The following modelled results (Table 2) experimentally validated were obtained, showing the trend of increase in inhibition efficiency with increasing concentrations of the inhibitor. This trend is graphically displayed in Figure 5.

(4a) $$\begin{array}{c} \text{IE}\left(\%\right)=+92.61-2.32A-18.34B+1.63AB+2.23A^{2}+33.09B^{2}-1.63A^{2}B+0.6540AB^{2}+1.13A^{3}+66.52B^{3}-1.06A^{2}B^{2}-0.2587A^{3}B-2.31AB^{3}-1.42A^{4}-78.02B^{4}, \end{array}$$

(4b) $$\begin{array}{c} \text{CR}\left(\frac{\text{mm}}{\text{yr}}\right)=+0.0436-0.0772A+0.4081B+0.0875AB+0.5997A^{2}-0.6817B^{2}-0.2054A^{2}B-0.2842AB^{2}+0.1402A^{3}-1.03B^{3}+0.3540A^{2}B^{2}-0.2537A^{3}B+0.3870AB^{3}-0.5706A^{4}+1.20B^{4}, \end{array}$$

(4c) $$\begin{array}{c} \text{WL}\left(\text{g}\right)=-0.36014+0.616333A-1.87714B-0.863571AB+0.391905A^{2}+2.84754B^{2}. \end{array}$$

Figure 1 shows that the corrosion rate for the blank specimen initially increased rapidly; however, after 50 h of exposure, the corrosion rate decreased probably as a result of the passivation of the specimens by adherent corrosion products which were not readily removable from the surface of the specimens during cleaning [1, 35]. At 70 h immersion time, the rate of corrosion continued its upward surge as flaws within the adherent corrosion products were undermined to reveal the substrate to further corrosion activities in the acidic environment. With the addition of MBZ at various concentrations to the corroding systems, the corrosion rates were reduced to their barest minimum (Figure 2).

As displayed in Figure 2, MBZ was able maintain the low weight loss of the mild steel at a concentration of 0.5 g for exposure times ranging from 24 to 120 h. For 24 h exposure, the reduction in weight loss of mild steel while corroding freely was from 3 g to about 0.1 g on adding 0.5 g/l of the inhibitor to the corroding system. This massive reduction in weight loss was replicated for all concentrations of MBZ employed in this investigation. The reduction in the weight loss of the mild steel in the acid solution containing the MBZ can be attributed to the formation of a protective layer by the inhibitor molecules around the mild steel. It also points to the presence of heterocyclic nitrogen and lone pair of electron in the structure of the inhibitor that are readily available for adsorption on the surface of the mild steel [34, 36].

From Figure 3, it can be observed that the graphs followed a similar pattern to those of the gravimetric curves in Figure 2. This is expected because weight loss is a reflection of the magnitude of corrosion of the mild steel. Examination of the figure revealed that the corrosion rate of mild steel in dilute H_2_SO_4_ solution was reduced upon the introduction of MBZ into the corrosive medium. The extent of reduction in corrosion rate is seen to proceed gradually with further increase in the concentration of the inhibitor beyond 0.5 g/l, for all the immersion durations. This reduction in weight loss obtained in Figure 2 leads to a corresponding decrease in corrosion rates due to the corrosion retarding action of the studied inhibitor.

From Figure 4, the inhibition efficiency was high initially for all concentrations; however, a general slight decline was observed with exposure time as the inhibiting species adhered and probably desorbed from the metal substrate in competition/crowding with increasing exposure time [19, 37–39]. Considering the influence of the inhibitor concentration on the inhibition efficiency, a gradual increase could be noticed from 0.5 to 1.5 g/l.

In order to justify the general increase in the efficiency of MBZ at low concentrations, a model (equation (4a)) was developed and used to predict the inhibition efficiencies for concentrations of 0.05 g/l to 0.45 g/l. The predicted results presented in Table 2 were used to plot Figure 5. The modelled and predicted results of inhibition efficiencies for lower concentrations of the inhibitor succinctly show a definite trend of inhibition efficiency increasing with inhibitor concentration up to 0.5 g/l. However, between 0.5 g/l and 1.5 g/l, a slight variation was observed in the inhibition efficiencies as shown earlier in Figure 4. Thus, the inhibitor is predictably very effective at lower concentrations.

### 3.2. Electrochemical Measurements

#### 3.2.1. Potentiodynamic Polarization Measurements/Tafel Extrapolation

**Figure 6:**
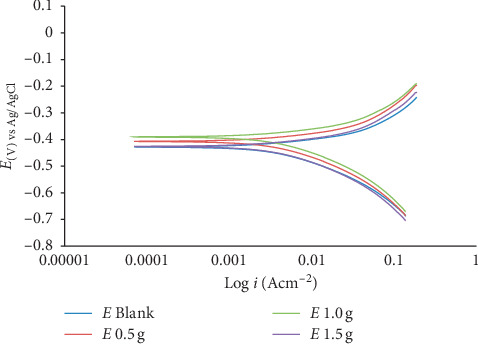
Potentiodynamic polarization curve of mild steel in dilute H_2_SO_4_ in the absence and presence of MBZ at 30°C.

**Figure 7:**
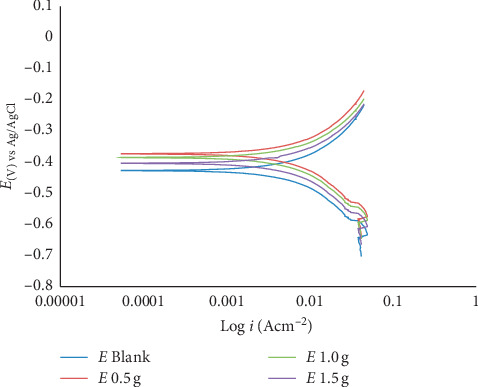
Potentiodynamic polarization curve of mild steel in dilute H_2_SO_4_ in the absence and presence of the corrosion inhibitor at 60°C.

The main purpose of polarization measurements was to find out the influence of the introduction of various concentrations of MBZ on the dissolution rate of mild steel as well as the corresponding cathodic reduction rate of hydrogen ion during the corrosion process. The polarization plots obtained for the corrosion of mild steel in dilute H_2_SO_4_ in the absence and presence of inhibitor at various concentrations are shown in Figures 6 and 7. The polarization curves indicated that the introduction of MBZ into H_2_SO_4_ had moderately pronounced effects on both the anodic and cathodic reactions. The Tafel extrapolation of the polarization curves gave the potentiodynamic parameters as described in Tables 3 and 4. From Tables 3 and 4, it could be observed that the introduction of MBZ shifted the *E*_corr_ significantly in the positive direction indicative of an anodic inhibition. A corrosion inhibitor may act as a cathodic, anodic, or mixed type, depending on the level of the displacement of the *E*_corr_. If the displacement in *E*_corr_ is greater than 85 mV with reference to *E*_corr_, the inhibitor may act as a cathodic or anodic type, and if the displacement is less than 85 mV, the inhibitor may be classified as a mixed type [12, 40]. It could be deduced from the polarization results that the MBZ behaved as mixed-type inhibitor. The displacement of *E*_corr_ observed in this study is less than 85 mV both at 30°C and 60°C.

The results of maximum corrosion inhibition efficiencies computed from gravimetric and the polarization parameters at both 30°C and 60°C were generally similar in numerical values which suggested that both methods complemented each other. The MBZ inhibitor protected the mild steel from corrosion in the aggressive solution with maximum inhibition efficiencies of 84.93% for 1.5 g MBZ in dilute H_2_SO_4_ at elevated temperature, whereas 99.05% was observed for 1.0 g MBZ at room temperature.

#### 3.2.2. Effect of Temperature and Mechanism of Adsorption

The rate of corrosion as expected increases with increase in temperature for it is generally known that for every 10°C rise in temperature, the rate of any reaction doubles. However, the efficiency of MBZ at 60°C declined to between about 35 to 85% for inhibitor concentrations of 0.5 g and 1.5 g, respectively, as against 96 and 97% for similar concentrations at room temperature. This observation can be ascribed to the rapid etching and desorption of inhibitor molecules from the surface of the substrate [2, 41–44], suggesting initial physical adsorption [45]. This view is corroborated by the findings of [19] and by [27]. Alternatively, an increase in inhibition efficiency and decrease in corrosion rate with rise in temperature is indicative of chemisorption mechanism. The decreased inhibition efficiencies and higher corrosion rates obtained for the test specimens at elevated temperature as suggested by other researchers [19, 22] were an indication that the critical concentration of anions required for the protection of mild steel in the corrosive media increases as the temperature increased.

**Figure 8:**
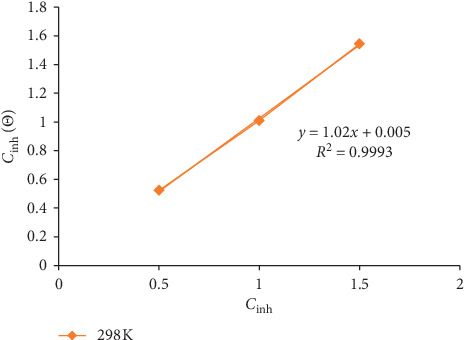
Langmuir adsorption isotherm for the adsorption of MBZ on mild steel in dilute H_2_SO_4._

In order to further obtain information about the mechanism of inhibition, the data obtained in the electrochemical (potentiodynamic polarization) investigation were further analysed using adsorption isotherms as described by [30]. Frumkin, Temkin, Freundlich, and Langmuir isotherms and their variations are the most frequently used isotherms to evaluate the mechanism of inhibition of corrosion rates. From the various plots, the Langmuir adsorption isotherm provided the best and most suitable description of the behaviour of MBZ with a linear graph and near unity slopes and correlation coefficient of 1.02 and 0.9993, respectively. The correlation coefficient (*R*^2^) value (0.3393) for the reaction at 60°C deviated significantly from unity, which also supported the deduction that the critical concentrations of anions required for the protection of metals in corrosive media increased as the temperature was increased. There was a slight slope deviation and correlation coefficients of the Langmuir plot from unity, which may be attributed to the interactions among adsorbed species on the metal surface and changes in the adsorption heat with increasing surface coverage [23, 24, 40]. The adsorption isotherm, Figure 8, was plotted for MBZ using the linear form of the Langmuir adsorption isotherm shown in equation (5):

(5) $$\begin{array}{c} \frac{C_{\text{inh}}}{\theta }=\frac{1}{K_{\text{ads}}}+C_{\text{inh}}, \end{array}$$

where *θ*, *K*_ads_, and *C*_inh_ indicated the degree of surface coverage, adsorption/desorption process equilibrium constant, and inhibitor concentration, respectively.

Employing equation (6), the change in adsorption Gibb's free energy (Δ*G*_ads_°) was obtained:

(6) $$\begin{array}{c} \Delta G_{\text{ads}}^{{}^{\circ}}=-RT\ln\left(55.5K_{\text{ads}}\right), \end{array}$$

where *R* is gas constant (8.314 kJ^−1^·mol^−1^), 55.5 is a constant that depicts the molar concentration (mol·L^−1^) of water in the solution, *K*_ads_ is the adsorption process equilibrium constant for the adsorption process, and *T* is the absolute temperature.  Table 5 presents the computed values of *K*_ads_ and Δ*G*_ads_° for the inhibitor at various temperatures and inhibitor concentrations. As a result of the effective and spontaneous adsorption of the MBZ molecules to the surface of mild steel in H_2_SO_4_, *K*_ads_ values were high and negative values were obtained for Δ*G*_ads_° Through the magnitude of Δ*G*_ads_°, the adsorptive nature was determined. However, for physical adsorption mechanism, values of about −20 kJ· mol^−1^ and less are associated, while values of −40 kJ· mol^−1^ and above are usually considered to be chemisorption [13, 24, 28, 30]. The results in Table 5 revealed that the computed values of Δ*G*_ads_° for the inhibitor ranged between −17.76 to −21.48 kJ·mol^−1^ and −11.33 to −15.25 kJ·mol^−1^ at 303 K and 333 K, respectively. This implied that the adsorption process of the inhibitor was by physiosorption. The values obtained for Δ*G*_ads_° and *K*_ads_ were observed to decrease with increase in temperature and inhibitor concentration which is corroborated by the findings of [31].

### 3.3. Surface Morphology

**Figure 9:**
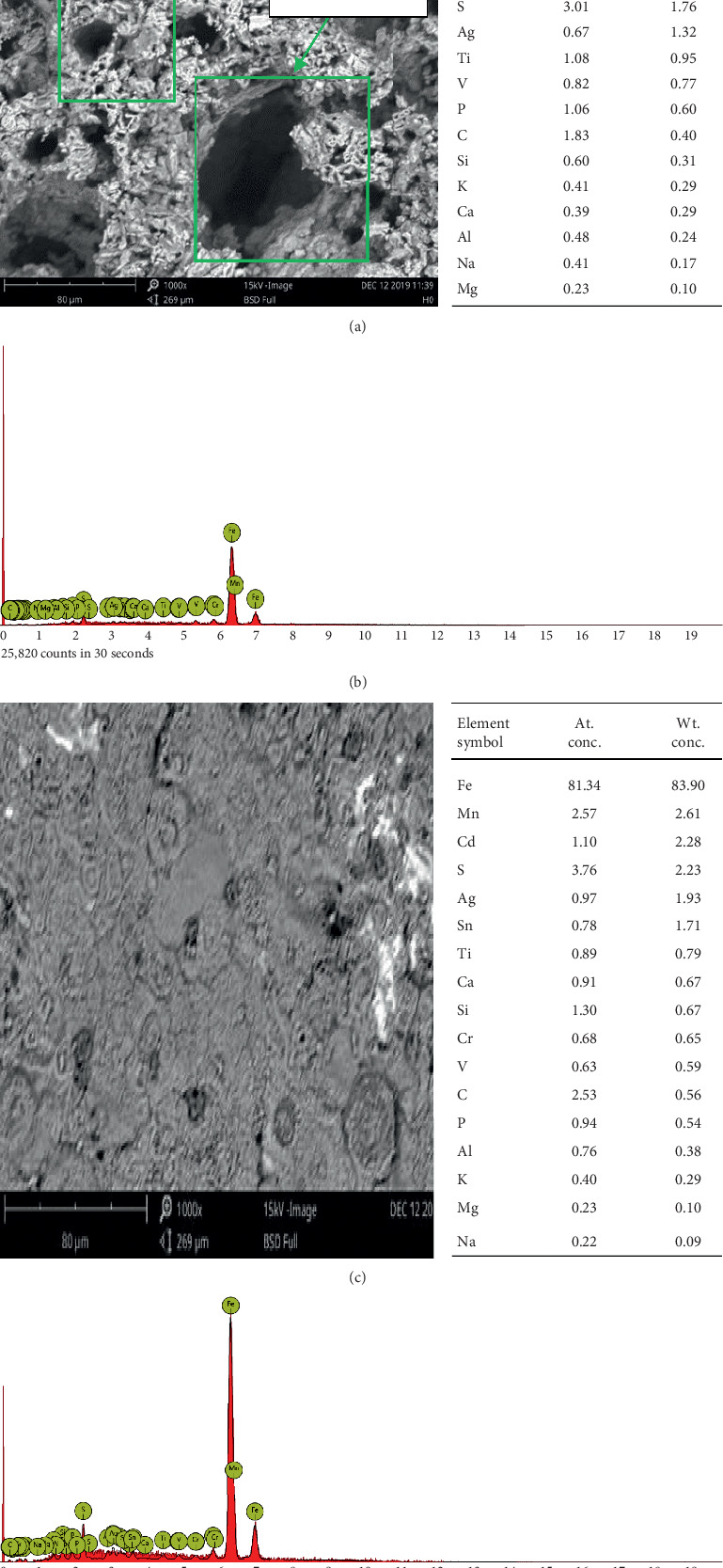
SEM micrograph of mild steel coupon with the EDX analysis data after 4 days immersion in H_2_SO_4_ without MBZ inhibitor and containing 1.5 g of MBZ. EDX spectra of mild steel coupon after 4 days immersion in a 1.5 M H_2_SO_4_ solution containing 1.5 g of MBZ inhibitor.

From the scanning electron microscopy (SEM) images shown in Figure 9(a), a number of wide and deep corrosion pits, marked with square box, can be observed on the mild steel surface besfore the introduction of MBZ. Sulphuric acid at low pH is known to cause aggravated damages to mild steel; however, in the presence of MBZ inhibitor, there was a significant protection of the specimen leading to a relatively smooth morphology. Figures 9(b) and 9(d) show the EDX spectra, while Figures 9(a) and 9(c) also have the tabulated EDX results showing the elemental composition of the mild steel coupon.

The SEM micrograph displayed in Figure 9(c) revealed some corrosion products, marked Y, which are probable stifled pitting corrosion initiated regions, in the presence of MBZ inhibitor.

The metallic ions indicated in the EDX analyses in Figures 9(a) and 9(c) and those indicated in the spectra of Figures 9(b) and 9(d) are mostly derived from the mild steel specimen, whereas sulphur, sodium, and some other species like phosphorus may have been derived from the sulphuric acid employed in this investigation.

## 4. Conclusions

The heteroatoms being the active centre in the inhibitor moiety effectively reduced the corrosion rate of mild steel in the dilute sulphuric acid employed in this investigation. Tafel extrapolation from the polarization curves showed that MBZ performed as a mixed inhibitor such that it reduced the rates of both the anodic and cathodic reactions. The percentage inhibition on the corrosion rate of mild steel in dilute sulphuric acid increased with increase in the concentration of MBZ, whereas it decreased with increase in the temperature regimes employed in this study, which is indicative of physiosorption mechanism. From thermodynamics considerations, the adsorption process followed the Langmuir adsorption isotherm. The surface morphologies, as revealed from SEM before and after addition of MBZ as corrosion inhibitor, support and give credence to the gravimetric and electrochemical tests.

## Figures and Tables

**Table 1:** Weight loss (g) of mebendazole in dilute H_2_SO_4_ at various inhibitor concentrations and exposure times.

Exposure time (hrs)	Corrosion properties	Inhibitor concentrations (g/l)			
		Blank	0.5	1.0	1.5
24	WL (g)	2.96	0.15	0.10	0.13
	CR (mm/yr)	2.19	0.11	0.07	0.01
	*η* (%)	—	94.98	96.62	95.61
	Φ	—	0.95	0.97	0.96

48	WL (g)	5.64	0.22	0.27	0.30
	CR (mm/yr)	2.09	0.08	0.10	0.11
	*η* (%)	—	94.68	96.28	96.10
	Φ	—	0.95	0.96	0.96

72	WL (g)	3.37	0.35	0.19	0.20
	CR (mm/yr)	0.83	0.09	0.05	0.05
	*η* (%)	—	89.61	94.36	94.07
	Φ	—	0.90	0.94	0.94

96	WL (g)	6.07	0.54	0.36	0.36
	CR (mm/yr)	1.12	0.10	0.07	0.07
	*η* (%)	—	91.10	94.07	94.07
	Φ	—	0.91	0.94	0.94

120	WL (g)	9.31	0.46	0.68	0.78
	CR (mm/yr)	1.38	0.07	0.10	0.12
	*η* (%)	—	91.62	95.70	95.06
	Φ	—	0.92	0.96	0.95

**Table 2:** Predicted weight loss (g) results of mild steel in the presence of small quantities of MBZ in dilute H_2_SO_4_.

Exposure time (h)	Inh. conc. (g/l)	Weight loss (g)	Corrosion rate (mm/yr)	Inhibition efficiency (%)
24	0.05	3.01	1.89	16.86
48	0.05	3.44	1.58	16.60
72	0.05	4.07	1.13	15.41
96	0.05	4.89	1.28	15.32
120	0.05	5.90	1.95	16.27
24	0.1	2.76	1.60	31.32
48	0.1	3.17	1.34	30.93
72	0.1	3.78	0.93	29.61
96	0.1	4.58	1.11	29.42
120	0.1	5.58	1.77	30.26
24	0.2	2.28	1.11	55.04
48	0.2	2.65	0.94	54.42
72	0.2	3.23	0.60	52.91
96	0.2	3.99	0.82	52.52
120	0.2	4.96	1.47	53.16
24	0.3	1.83	0.73	72.71
48	0.3	2.17	0.65	71.92
72	0.3	2.71	0.37	70.24
96	0.3	3.45	0.62	69.70
120	0.3	4.37	1.26	70.17
24	0.4	1.42	0.45	85.31
48	0.4	1.73	0.43	84.39
72	0.4	2.23	0.21	82.60
96	0.4	2.93	0.49	81.93
120	0.4	3.83	1.11	82.26

**Table 3:** Potentiodynamic polarization parameters at 30°C.

Inhibitor conc. (g)	*I*_corr_ (*µ*A)	*E*_corr_ (mV)	Corrosion rate (mm/yr)	Inhibitor efficiency (%)	Cathodic beta (mV)	Anodic beta (mV)
0.0	−6.58 mA	−429.75	48.61	—	182.00	104.32
0.5	−286.02	−409.55	2.11	95.65	136.93	84.26
1.0	−62.22	−407.58	0.46	99.05	110.80	57.01
1.5	−182.34	−442.46	1.35	97.23	99.91	134.25

**Table 4:** Potentiodynamic polarization parameters at 60°C.

Inhibitor conc. (g)	*I*_corr_ (mA)	*E*_corr_ (mV)	Corrosion rate (mm/yr)	Inhibitor efficiency (%)	Cathodic beta (mV)	Anodic beta (mV)
0.0	−48.08	−427.50	355.32	—	1.47 V	971.45
0.5	−31.23	−384.01	230.81	35.04	469.28	421.72
1.0	−8.82	−385.40	65.22	81.65	224.48	108.94
1.5	−7.24	−396.94	53.54	84.93	222.56	113.70

**Table 5:** Gibbs free energy, surface coverage, and equilibrium constant of adsorption for MBZ inhibitor in 1.5 M H_2_SO_4_.

Temperature (K)	Inhibitor conc. (g/l)	Δ*G*_ads_° (kJ·mol^−1^)	*K*_ads_ (mol^−1^)	Surface coverage
303	0.5	−19.33	44.00	0.96
	1.0	−21.48	104.71	0.99
	1.5	−17.76	23.38	0.97
333	0.5	−11.33	1.08	0.35
	1.0	−15.25	4.45	0.82
	1.5	−14.78	3.76	0.85

## Data Availability

The data used to support the findings of this study are included within the article.
